# Splenic pathologies: A pictorial review of common, wandering, twisting and rare presentations

**DOI:** 10.4102/sajr.v30i1.3366

**Published:** 2026-03-30

**Authors:** Audrey R. Rumhumha, Phumudzo E. Mphephu, Lungile M. Gabuza

**Affiliations:** 1 Department of Diagnostic Radiology, School of Clinical Medicine, Faculty of Health Sciences, University of the Witwatersrand, Johannesburg, South Africa

**Keywords:** wandering spleen, torsion, infarction, hydatid cyst, splenic pathology, pictorial review, radiology

## Abstract

Splenic pathologies, often overlooked in abdominal imaging, encompass a broad spectrum of imaging appearances and diagnostic challenges, including congenital anomalies, benign cysts, infarcts, trauma, vascular lesions, infections and neoplasms. Recognising these entities is crucial to distinguish incidental benign findings from potentially life-threatening conditions. This pictorial review illustrates the spectrum through 20 cases, including a rare case of a wandering spleen complicated by torsion and auto-infarction that demonstrates the classical ‘whorled pedicle’ and ‘twisted vascular pedicle’ signs. Ultrasound, CT, MRI and PET-CT images demonstrate typical and atypical features, emphasising key differentiating points and teaching pearls. Although not exhaustive, this overview underscores that closer scrutiny of the spleen is rewarding: a structured approach focusing on lesion location, enhancement patterns and clinical context improves diagnostic accuracy and guides timely management.

## Introduction

Despite its central haematologic and immunologic functions, the spleen remains underappreciated, and its pathologies are often overlooked because of subtle imaging features, low incidence or incidental presentation. This review revisits splenic disease through illustrative cases, emphasising imaging appearances, differentiating features and common diagnostic pitfalls. By integrating multimodality findings, we aim to strengthen radiologists’ confidence in assessing this deceptively modest yet diagnostically rich organ. Building on this foundation, a comprehensive overview spanning congenital, vascular, infectious, haematologic and neoplastic entities is presented. The review highlights splenic pathology through a multimodality lens, integrating radiographic, sonographic, CT, MRI and PET-CT appearances to demonstrate cross-sectional correlation and broaden the diagnostic perspective.

## Imaging overview

Assessment of splenic pathology benefits from a multimodality approach. Ultrasound serves as an excellent screening tool, particularly for cystic and vascular lesions. CT remains the cornerstone for evaluating trauma, infarction, infection and mass lesions. MRI offers superior soft tissue contrast, and is useful for characterising cystic and infiltrative processes. Doppler and contrast-enhanced ultrasound (CEUS) can aid in assessing vascular compromise, particularly in cases of suspected torsion or infarction. PET-CT offers complementary functional information and is particularly valuable in lymphoma, metastases and inflammatory conditions.

Although limited in clinical practice, plain radiography may still provide useful clues when calcifications are present. A discrete calcified focus projected over the splenic region, as demonstrated in one of the cases in the series, can suggest chronic or benign pathology and appropriately direct further cross-sectional imaging.

## Spectrum of splenic pathologies

### Pseudocyst or epidermoid cyst

**FIGURE 1:**
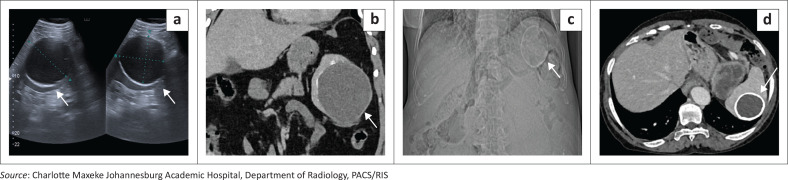
(a, b) Benign non-parasitic splenic cyst in a patient evaluated for a left upper quadrant mass. (a) Ultrasound demonstrates a well-circumscribed hypoechoic cystic lesion without internal septations or mural nodularity, with a thin rim of peripheral calcification (arrows). (b) Coronal contrast-enhanced CT in the same patient confirms a rounded, homogeneous fluid-attenuation cyst within the splenic parenchyma, characterised by a thin discontinuous curvilinear calcified rim (arrow). No internal septations, mural nodules, enhancing solid components or perisplenic inflammatory change are identified. (c, d) Benign non-parasitic splenic cyst identified incidentally in a 67-year-old male undergoing evaluation for renal calculi. (c) Scanogram (AP radiograph) demonstrates a well-circumscribed rounded lesion in the region of the spleen with a conspicuous peripheral calcified rim (arrow). (d) Axial contrast-enhanced CT confirms a cystic splenic lesion with a thick continuous calcified wall and homogeneous fluid attenuation, without internal septations, mural nodules or enhancing solid components (arrow).

Non-parasitic splenic cysts are rare benign lesions, typically classified as true epithelial (epidermoid) cysts or post-traumatic pseudocysts. Imaging differentiation can be challenging, especially when calcifications are present. On CT, both appear as well-defined hypodense lesions; pseudocysts more often show thicker, irregular rim calcification and a history of prior injury, whereas epidermoid cysts have thin, smooth walls and occur in younger patients (Figure 1). When features overlap, the term ‘benign non-parasitic splenic cyst’ is appropriate.

**Key teaching point:** The absence of internal septations or daughter membranes helps distinguish benign non-parasitic splenic cysts (including pseudocysts and epidermoid cysts) from parasitic cysts such as hydatid disease on imaging.^1^

### Splenic haemangioma or hamartoma

**FIGURE 2:**
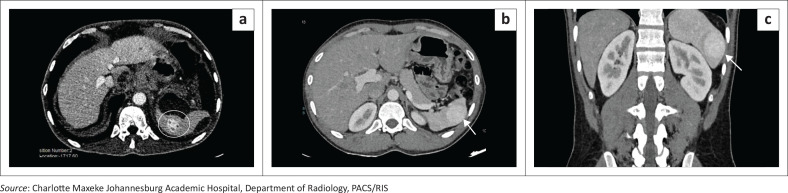
Splenic haemangioma and hamartoma identified incidentally in two patients. (a) Axial contrast-enhanced CT demonstrates a well-defined splenic lesion with characteristic discontinuous peripheral nodular enhancement and progressive centripetal fill-in, consistent with a splenic haemangioma (circle). (b, c) Axial and coronal contrast-enhanced CT images in a different patient demonstrate a well-circumscribed rounded splenic lesion showing relatively homogeneous enhancement (arrows), which may represent either a haemangioma or hamartoma. There is no associated splenomegaly, calcification or surrounding inflammatory change.

Splenic haemangiomas are the most common benign primary splenic neoplasm often showing peripheral nodular discontinuous enhancement with progressive centripetal fill-in, paralleling hepatic haemangiomas. Imaging overlap with hamartoma can occur; both entities can present as solitary, well-defined splenic masses with variable enhancement patterns (Figure 2a–c), occasionally making the distinction on imaging alone challenging. Histopathologic correlation is often required for definitive diagnosis.^2^

### Wandering spleen with torsion and auto-infarction

**FIGURE 3:**
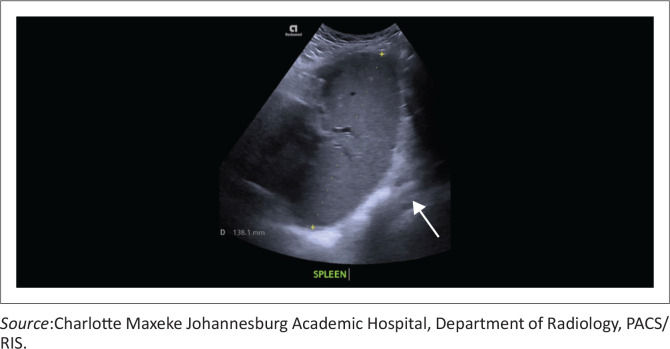
Greyscale ultrasound demonstrating splenomegaly, with the spleen in the left upper quadrant measuring 13.8 cm in craniocaudal dimension.

**FIGURE 4:**
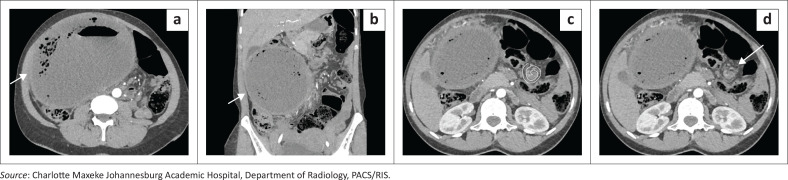
Splenic torsion in a 28-year-old female with known splenomegaly presenting with acute abdominal pain. (a, b) Axial and coronal contrast-enhanced CT images demonstrate absence of the spleen from its normal left upper quadrant location and the presence of an ectopic spleen within the lower abdomen (arrows). (c, d) The twisted vascular pedicle (“whirlpool sign”) is demonstrated (c and arrow in d). The spleen shows complete absence of enhancement with a central hypodense region containing internal air locules, consistent with infarction complicated by superimposed infection.

Torsion is a rare but critical complication of a wandering spleen, resulting from twisting of the elongated vascular pedicle on its axis. The torsion may be partial or complete, with consequent venous congestion, splenic enlargement (Figure 3), and, if prolonged, parenchymal infarction (Figure 4a and b). On imaging, the ‘whorled’ or ‘whirlpool’ sign, characterised by concentric twisting of the splenic vessels and pedicle (Figure 4c and d), is the key diagnostic feature on both Doppler ultrasound and contrast-enhanced CT or MRI. Additional findings may include a displaced spleen, variable splenic enhancement depending on the degree of vascular compromise, and associated ascites. Timely recognition of these imaging features enables splenopexy in salvageable cases; delayed diagnosis often necessitates splenectomy.^3^

**Key teaching point**: Early recognition of the ‘whirlpool sign’ is crucial to prevent irreversible infarction.^3^

### Ruptured hydatid cyst

**FIGURE 5:**
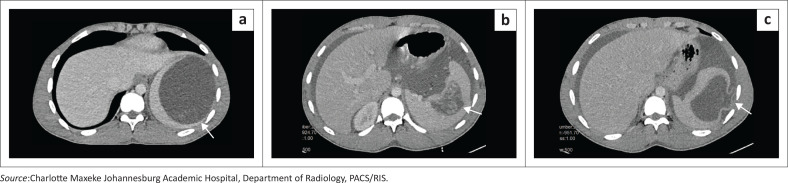
Ruptured splenic hydatid cyst in a 16-year-old patient presenting with acute abdominal pain. (a) Axial contrast-enhanced CT demonstrates a unilocular cystic splenic lesion with hyperdense ill-defined dependent internal contents (arrow). (b, c) Additional axial CT images demonstrate detached internal membranes within the cyst, characteristic of hydatid disease (arrow in b). Focal thinning and irregularity of the adjacent splenic parenchyma suggests the site of rupture (arrow in c), with associated large-volume ascites and no peritoneal deposits or additional sites of disease.

Splenic hydatid disease is a rare manifestation of *Echinococcus granulosus*, accounting for < 2% of abdominal echinococcosis.^4^ Isolated splenic involvement typically reflects haematogenous seeding. Imaging features include a well-defined cystic lesion with daughter cysts, floating or detached membranes (‘water-lily sign’)^4^ (Figure 5a–c) and curvilinear or rim calcification, the latter two findings being highly specific.

**FIGURE 6:**
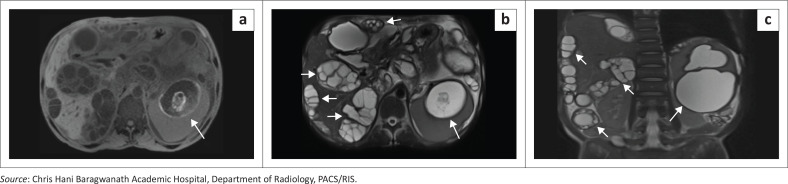
Disseminated hydatid disease in an adult patient. (a) Axial T1-weighted MRI demonstrates diffuse peritoneal hydatidosis with multiple peritoneal lesions. The splenic lesion demonstrates areas of intrinsic T1 hyperintensity and a peripheral hyperintense rim (arrow). (b) Axial T2-weighted HASTE sequence demonstrates multiloculated peritoneal hydatid cysts and a well-circumscribed splenic hydatid cyst with peripheral signal dropout corresponding to a calcified rim and internal multiseptated daughter cysts (arrow). (c) Coronal T2-weighted HASTE image confirms the splenic origin of the dominant lesion and demonstrates additional peritoneal and hepatic hydatid lesions (arrows).

In contrast, multiorgan hydatid disease reflects more extensive dissemination with concurrent hepatic, peritoneal and splenic involvement (Figure 6a–c). Liver cysts are the most common manifestations and may act as the source of peritoneal seeding through spontaneous rupture or iatrogenic spread.^5^ Peritoneal implants often present as multiple thin-walled cysts scattered along serosal surfaces.

Across both isolated and disseminated forms, complications such as secondary infection, rupture, anaphylaxis, or mass effect dictate the urgency of management. CT and MRI play crucial roles not only in diagnosis but also in classification and surgical planning.^5^

**Key teaching point:** It is essential to identify the ‘water-lily sign’ and assess for vascular complications as well as peritoneal seeding.^4^

### Vascular lesions of the spleen

**FIGURE 7:**
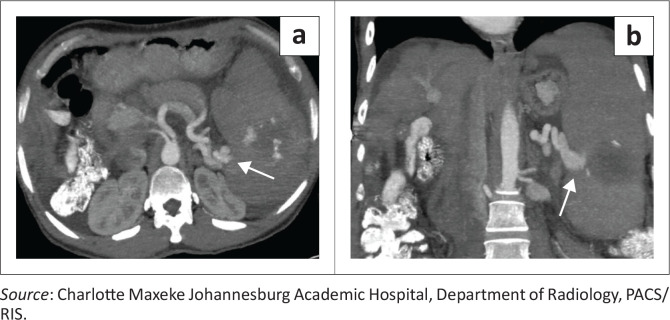
Splenic venous varix in a 44-year-old woman with portal hypertension who developed an enlarging splenic venous pseudoaneurysm that subsequently ruptured. (a, b) Axial and coronal contrast-enhanced CT images demonstrate a dilated tortuous splenic venous varix (arrows) with an associated intrasplenic haematoma. The splenic vein demonstrates early opacification during the arterial phase and the adjacent splenic artery is seen in close proximity, raising suspicion for an underlying vascular malformation.

Venous and arterial vascular abnormalities of the spleen represent a rare and often under-recognised subset of splenic pathology. In this review, two illustrative cases highlight this spectrum, with the first demonstrating a venous abnormality and the second an arterial lesion. The initial case illustrates a splenic venous pseudoaneurysm (also referred to as a splenic vein varix), an exceptionally uncommon entity most often described in association with trauma, pancreatitis, infection or adjacent inflammatory processes. On cross-sectional imaging, the lesion appears as a focal, contrast-opacified venous outpouching contiguous with the splenic vein, with well-defined margins and enhancement characteristics concordant with the venous system on arterial and portal venous phases. Recognition is critical, as splenic venous pseudoaneurysms carry a significant risk of rupture, haemorrhage or thrombosis and may necessitate urgent endovascular or surgical management depending on clinical stability (Figure 7a and b).

**Key teaching point:** Pseudoaneurysms may rupture even when small; early detection and vascular intervention are critical.^6^

**FIGURE 8:**
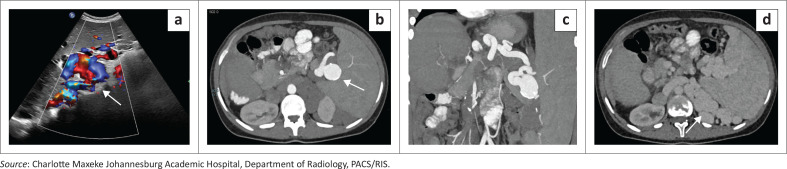
Splenic artery aneurysm in a 19-year-old patient with a complex medical history including a prior Kasai procedure and longstanding portal hypertension.(a) Colour Doppler ultrasound demonstrates a well-defined vascular lesion with the characteristic “yin–yang” bidirectional flow pattern consistent with an aneurysmal sac (arrows). (b) Axial arterial-phase contrast-enhanced CT shows a rounded saccular dilatation of the splenic artery (arrow). (c) Coronal reformatted CT confirms the aneurysm along the course of the splenic artery. (d) Axial contrast-enhanced CT demonstrates multiple serpiginous enhancing perisplenic varices (arrow) consistent with portal hypertension related collateralisation.

Splenic artery aneurysms (Figure 8a–d) are the most common visceral arterial aneurysms and are more prevalent in conditions associated with increased splenic arterial flow, such as portal hypertension and pregnancy. Although often asymptomatic, they pose a significant risk of rupture, particularly when larger than 3 cm, and early identification is essential.^6^ The accompanying variceal network and enlarged spleen not only contextualise the underlying pathophysiology but also underscore the importance of detailed vascular assessment in patients with portal hypertensive states.

**Key teaching point:** There are diverse vascular manifestations that may involve the spleen, and there is a need for careful evaluation of both arterial and venous structures during abdominal imaging.

### Polysplenia (heterotaxy syndrome)

**FIGURE 9:**
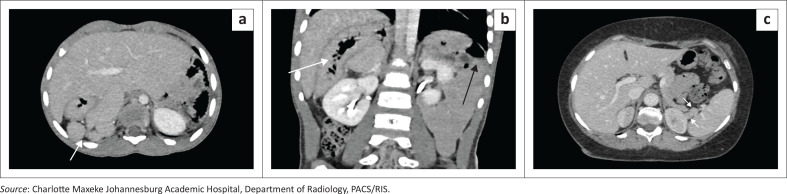
Heterotaxy syndrome identified incidentally in a 6-year-old trauma patient. (a, b) Axial and coronal contrast-enhanced CT images demonstrate multiple splenules (white arrow in a) in a patient with polysplenia associated with heterotaxy syndrome, with an empty splenic fossa (black arrow) and a right-sided stomach (white arrow in b). (c) Axial contrast-enhanced CT in a different patient demonstrates multiple small splenules adjacent to a dominant spleen (arrows), representing a normal anatomical variant (splenunculi).

Polysplenia is part of the heterotaxy (left isomerism) spectrum, characterised by multiple splenunculi and abnormal thoracoabdominal arrangement (e.g. interrupted inferior vena cava with azygos continuation and midline liver). Identification of multiple splenunculi and associated anomalies on CT/MRI is diagnostic; evaluation of cardiovascular anomalies is essential for patient management and surgical planning^7^ (Figure 9a–c).

**Key teaching point:** Evaluate for associated cardiovascular and visceral anomalies.^7^

### Haemoglobinopathy – Sickle cell disease

**FIGURE 10:**
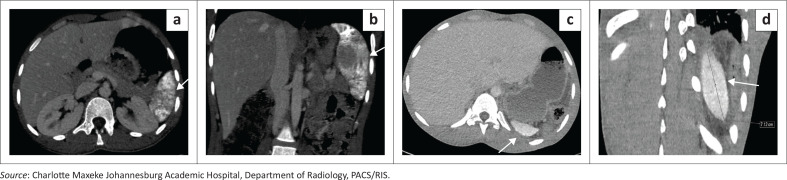
Splenic findings in sickle cell disease in a 24-year-old male presenting with jaundice and anaemia and in a separate patient with known sickle cell disease presenting during a chest crisis. (a, b) Axial contrast-enhanced CT images demonstrate coarse patchy calcifications within a normal-sized spleen (arrows), reflecting repeated splenic infarctions. (c, d) Axial contrast-enhanced CT images in a different patient demonstrate a diffusely hyperdense heavily calcified small spleen consistent with autosplenectomy (arrows).

In sickle cell disease, repeated vaso-occlusive infarctions lead to progressive splenic fibrosis, atrophy, and eventual ‘autosplenectomy’. Early childhood may show splenomegaly from sequestration and extramedullary haematopoiesis, but chronic infarction results in a small, calcified, non-functioning spleen.^8^ CT demonstrates marked atrophy and dense calcification (Figure 10a–d), while MRI shows diffusely low signal from fibrosis and hemosiderin. Recognising these features is key, as functional asplenia increases the risk of overwhelming post-splenectomy infection.

**Key teaching point:** Splenic calcification in a young patient strongly suggests haemoglobinopathy.^9^

### Splenic microabscesses (disseminated tuberculosis)

**FIGURE 11:**
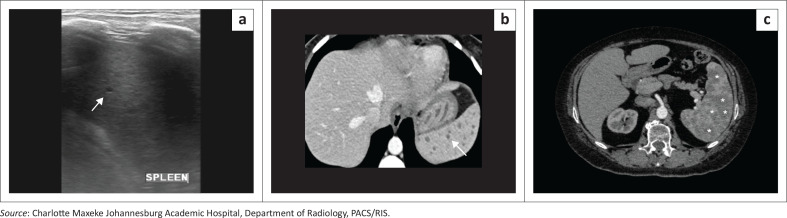
Granulomatous splenic disease in a patient with tuberculosis and in a separate patient with sarcoidosis. (a) Ultrasound and (b) axial contrast-enhanced CT images demonstrate multiple tiny hypodense splenic nodules in a patient with tuberculosis (arrows), consistent with splenic microabscesses. (c) Axial contrast-enhanced CT in a different patient demonstrates diffuse granulomatous infiltration of the spleen with innumerable well-circumscribed hypodense nodules (stars), consistent with sarcoidosis.

Granulomatous diseases such as tuberculosis and sarcoidosis commonly involve the spleen as part of multi-system disease processes, producing overlapping imaging features that may mimic neoplastic or infectious aetiologies. In splenic tuberculosis, lesions may be solitary or multiple, often hypodense on CT, with caseating necrosis leading to central low attenuation and peripheral rim enhancement (Figure 11a and b); chronic lesions may calcify.^10^ Sarcoidosis, by contrast, typically manifests as diffuse splenomegaly or numerous tiny hypodense nodules that are non-enhancing and frequently accompanied by hepatic or nodal involvement (Figure 11c). On MRI, both entities demonstrate low T1 and variably low T2 signal intensities depending on the fibrotic content, with minimal enhancement. Other granulomatous or inflammatory conditions such as histoplasmosis or brucellosis can appear similar,^10^ necessitating clinical correlation and supportive laboratory or histopathological confirmation. Awareness of these overlapping appearances helps avoid misdiagnosis of metastatic or lymphomatous disease, guiding appropriate medical rather than surgical management.

**Key teaching point:** Differentiating microabscesses in disseminated infection (tuberculosis, fungal) is based on clinical and systemic findings.^10^

### Splenic lymphoma

**FIGURE 12:**
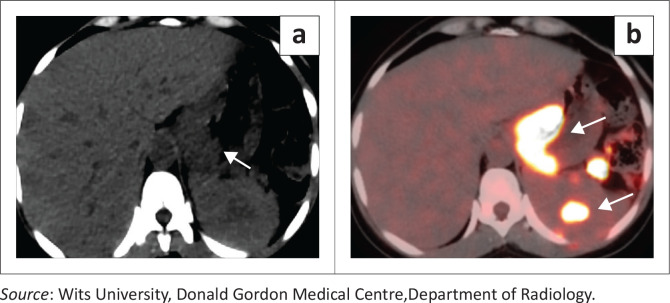
Histologically confirmed large B-cell non-Hodgkin lymphoma in an adult patient. (a) Non-contrast attenuation-correction CT demonstrates focal thickening of the gastric wall with associated low-density splenic hilar lymph nodes (arrow). (b) Fused PET-CT image demonstrates fluorodeoxyglucose (FDG) uptake within the thickened gastric wall, splenic hilar lymph nodes and a focal splenic lesion (arrows), consistent with disseminated B-cell lymphoma.

**FIGURE 13:**
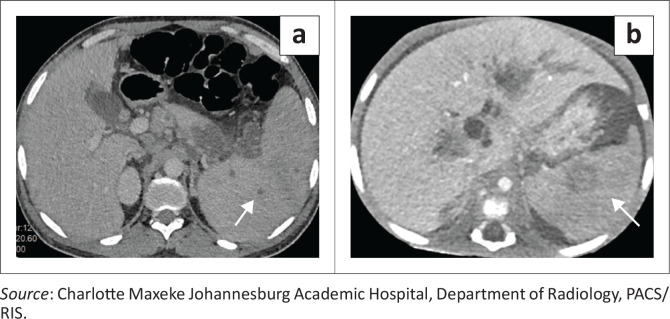
Hodgkin lymphoma in an adult female and Burkitt lymphoma in a 2-year-old male. (a) Axial contrast-enhanced CT demonstrates multiple rounded hypodense lesions scattered throughout the splenic parenchyma (arrow) with associated splenomegaly. Additional hypodense soft-tissue masses involving the pancreatic tail and perisplenic region likely represent direct extension from adjacent splenic or nodal disease. (b) Axial contrast-enhanced CT in a different patient demonstrates ill-defined nodular splenic infiltration (arrow).

In Hodgkin lymphoma, splenic involvement is a common manifestation of advanced disease and typically occurs through haematogenous or contiguous spread. On CT, the spleen may appear enlarged with or without discrete hypodense nodules. The nodules are often well-circumscribed, non-enhancing, and homogeneous, reflecting lymphomatous infiltration (Figure 12a–b).^10^ The imaging appearance of multiple rounded hypoattenuating lesions with splenomegaly in a patient with known Hodgkin lymphoma is characteristic (Figure 13a–b) and should not be mistaken for metastatic disease or infection.

### Splenic metastases

**FIGURE 14:**
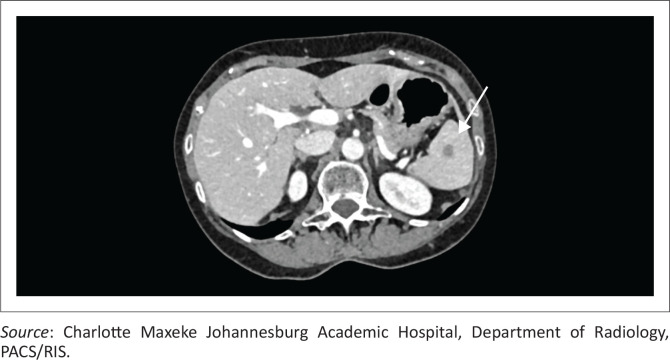
Splenic metastasis in a 44-year-old patient with primary breast carcinoma. Axial contrast-enhanced CT demonstrates a rounded hypodense lesion within the splenic parenchyma (arrow), consistent with metastatic disease.

Splenic metastases are uncommon and typically seen with disseminated disease (melanoma, breast, lung, ovarian and colorectal).^11^ Lesions are usually hypodense on CT and variable on MRI; FDG-PET is typically avid. Their presence signifies advanced disease with prognostic implications (Figure 14).

**Key teaching point**: Consider splenic involvement in patients with disseminated malignancy.^11^

### Splenic lesion mimics

**FIGURE 15:**
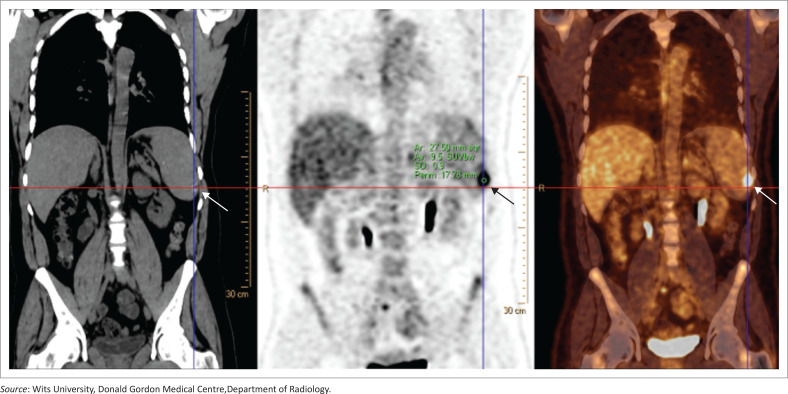
Peritoneal metastatic deposit mimicking a splenic lesion in a 37-year-old patient with a history of rectal adenocarcinoma. Coronal FDG PET demonstrates a peripheral stippled calcified focus with intense FDG uptake (SUV 9.5). Multiplanar reformats (not shown) confirmed that the abnormality was not intraparenchymal but instead located along the capsular surface of the spleen.

Several extra-splenic processes may project onto or above the spleen, creating the false impression of a primary splenic lesion.^12^ Peritoneal deposits, particularly in mucinous gastrointestinal malignancies, may adhere to the splenic capsule and mimic parenchymal disease, often showing calcifications or FDG avidity (Figure 15). Careful evaluation of the capsular versus intraparenchymal location on multiplanar imaging helps avoid misclassification of splenic involvement (Figure 15a–c).

**Key teaching point**: The importance of careful evaluation of lesion location and morphology to avoid misclassification of peritoneal deposits as primary splenic pathology.^12^

## Discussion

While many splenic lesions are incidental, distinguishing benign from malignant conditions remains essential. Radiologists should maintain a high index of suspicion when encountering atypical splenic morphology, especially in trauma, systemic infection, or known malignancy. A systematic approach considering location, enhancement pattern, and associated findings enhances diagnostic accuracy.^13^

Cross-sectional imaging is central to splenic evaluation. CT remains the primary modality because of its rapid acquisition, spatial resolution, and ability to evaluate enhancement patterns during arterial and portal venous phases. MRI, with its superior soft-tissue contrast, aids in differentiating solid from cystic lesions, while DWI enhances detection of microabscesses and metastases. CEUS has emerged as a valuable problem-solving tool, particularly in differentiating benign vascular lesions such as haemangiomas from malignant deposits or abscesses.^14^ When approaching a splenic lesion, three features are diagnostically pivotal: (1) enhancement characteristics; (2) multiplicity or distribution; and (3) clinical context.^15^

A comprehensive review of splenic pathology enhances diagnostic vigilance. Radiologists are encouraged to regard the spleen not as a passive organ but as an active participant in systemic disease. Table 1 summarises the spectrum of splenic pathologies.

**TABLE 1:** Summary of splenic pathologies and key imaging features.

Condition	Preferred modality	Key imaging features	Teaching points
Epidermoid (true) splenic cyst	US, CT	Thin-walled unilocular cyst; anechoic; non-enhancing; may show mural calcification	No daughter cysts or membranes; helps differentiate from hydatid disease
Post-traumatic pseudocyst	CT, US	Irregular wall; internal debris; peripheral calcification	Sequela of haematoma; lacks epithelial lining
Haemangioma	US, CT, MRI	Well-circumscribed lesion; peripheral nodular discontinuous enhancement with progressive centripetal fill-in; occasional cystic degeneration or calcification	Most common benign splenic tumour; closely mimics hamartoma. Differentiation relies on enhancement pattern but mostly histopathological
Wandering spleen with torsion	CT, Doppler	Ectopic spleen; twisted pedicle (‘whirl sign’); absent enhancement when infarcted	Early recognition prevents infarction; splenopexy if viable
Hydatid cyst	CT, US, MRI	Multiloculated cyst with internal membranes (‘water-lily sign’); daughter cysts; calcified rim; free fluid if ruptured	Risk of anaphylaxis or peritoneal seeding; assess for hepatic and peritoneal involvement
Splenic artery or vein pseudoaneurysm	CT Angiography, Doppler	Round enhancing lesion; contrast extravasation possible; irregular wall	Often post-trauma or pancreatitis; urgent vascular intervention may be required
Polysplenia (heterotaxy)	CT, MRI	Multiple splenules; left isomerism; associated IVC interruption	Look for associated cardiac and visceral anomalies
Splenic metastases	CT, MRI, PET-CT	Multiple low-attenuation lesions; variable enhancement; FDG-avid	Common primaries include melanoma, breast, lung
Haemoglobinopathy (sickle cell disease)	CT, US	Small, calcified, fibrotic spleen; autosplenectomy	Predisposes to infection with encapsulated organisms
Granulomatous disease (tuberculosis, sarcoidosis)	CT, MRI	Multiple small hypodense nodules; may be T2 hypointense; may calcify; variable restricted diffusion	Mimics fungal infection or metastases; evaluate systemic involvement
Splenic infarction	CT, MRI	Wedge-shaped peripheral non-enhancing areas	Associated with emboli, haematologic disorders, torsion
Lymphoma	CT, MRI, PET-CT	Splenomegaly or multiple hypodense nodules; FDG-avid	Most common splenic malignancy; PET important for staging
Splenic abscess	CT, US	Rim-enhancing hypodense lesion; may contain gas	Common in immunosuppressed patients; differentiate from infarct
Accessory spleen	CT, MRI	Round nodule with enhancement identical to spleen	Mimics lymphadenopathy or metastasis; confirm with heat-damaged Red blood cell (RBC) scan
Mimics of splenic lesions	CT, MRI, PET-CT	Peritoneal implants, adjacent gastric/colonic masses, Left upper quadrant (LUQ) nodes, granulomatous calcifications	Correlate with metabolic activity and anatomical relationships

## Conclusion

This review underscores the importance of recognising the spleen’s diverse imaging presentations, particularly rare entities such as torsion and hydatid disease, to improve diagnostic accuracy and patient outcomes. Ultimately, the radiologist’s willingness to wonder, to wander and to identify those rare twists can turn incidental findings into insightful diagnoses.

## Ethical considerations

All images were obtained from the institutional PACS archive and fully anonymised prior to inclusion. No patient identifiers are present. Formal ethics approval was not required for this retrospective educational review in accordance with institutional policy.
